# The Cadherin Protein Is Not Involved in Susceptibility to *Bacillus thuringiensis* Cry1Ab or Cry1Fa Toxins in *Spodoptera frugiperda*

**DOI:** 10.3390/toxins12060375

**Published:** 2020-06-06

**Authors:** Jianfeng Zhang, Minghui Jin, Yanchao Yang, Leilei Liu, Yongbo Yang, Isabel Gómez, Alejandra Bravo, Mario Soberón, Yutao Xiao, Kaiyu Liu

**Affiliations:** 1 Institute of Entomology, School of Life Sciences, Central China Normal University, Wuhan 430079, China; jianfengzhang@mails.ccnu.edu.cn (J.Z.); yangyanchao@mails.ccnu.edu.cn (Y.Y.); liulei@mails.ccnu.edu.cn (L.L.); yongboyang@mail.ccnu.edu.cn (Y.Y.); 2 Agricultural Genomics Institute at Shenzhen, Chinese Academy of Agricultural Sciences, Shenzhen 518120, China; jinminghui@caas.cn (M.J.); xiaoyutao@caas.cn (Y.X.); 3 Instituto de Biotecnología, Universidad Nacional Autónoma de México, Apdo. Postal 510-3, Cuernavaca 62250, Morelos, Mexico; isabelg@ibt.unam.mx (I.G.); bravo@ibt.unam.mx (A.B.); mario@ibt.unam.mx (M.S.)

**Keywords:** *Bacillus thuringiensis*, *Spodoptera frugiperda*, cadherin, Cry1Ab, Cry1Fa, mode of action of Cry toxin

## Abstract

It is well known that insect larval midgut cadherin protein serves as a receptor of *Bacillus thuringiensis* (Bt) crystal Cry1Ac or Cry1Ab toxins, since structural mutations and downregulation of *cad* gene expression are linked with resistance to Cry1Ac toxin in several lepidopteran insects. However, the role of *Spodoptera frugiperda* cadherin protein (SfCad) in the mode of action of Bt toxins remains elusive. Here, we investigated whether SfCad is involved in susceptibility to Cry1Ab or Cry1Fa toxins. *In vivo*, knockout of the *SfCad* gene by CRISPR/Cas 9 did not increase tolerance to either of these toxins in *S. frugiperda* larvae. *In vitro* cytotoxicity assays demonstrated that cultured insect TnHi5 cells expressing GFP-tagged SfCad did not increase susceptibility to activated Cry1Ab or Cry1Fa toxins. In contrast, expression of another well recognized Cry1A receptor in this cell line, the ABCC2 transporter, increased the toxicity of both Cry1Ab and Cry1Fa toxins, suggesting that SfABCC2 functions as a receptor of these toxins. Finally, we showed that the toxin-binding region of SfCad did not bind to activated Cry1Ab, Cry1Ac, nor Cry1Fa. All these results support that SfCad is not involved in the mode of action of Cry1Ab or Cry1Fa toxins in *S. frugiperda*.

## 1. Introduction

The crystal (Cry) toxins and vegetative insecticidal proteins (Vip) produced by *Bacillus thuringiensis* (Bt) bacteria are important biological tools for the control of insect pests and provide good protection for plants growth [1]. During sporulation, Bt bacteria accumulate Cry toxins in crystal inclusion bodies inside the mother cell, while the Vip proteins are secreted in the vegetative phase of growth [2,3]. The Bt toxin receptors, located on the larval midgut cells, play important roles in the toxicity of these Bt toxins. After ingestion of Bt crystal inclusions or Vip protein by the larvae, these proteins are dissolved under the alkaline conditions of the gut lumen, releasing protoxins that are activated by midgut proteases. The activated toxins bind to receptors, forming oligomers that insert into the cell membrane leading to pore formation, which results in death of the larvae [2,3]. The mode of action of Vip3Aa might be different from crystal toxins, since receptors for Vip3Aa are not shared with the Cry toxins [4,5,6,7].

In several lepidopteran insects, mutations in the *cadherin* gene (*cad*) are associated with resistance to Cry1Ac or Cry1Ab toxins [8,9,10,11,12]. The Cry1Ac toxin-binding region of *Helicoverpa armigera* cadherin (HaCad) and the membrane-proximal region of HaCad are required for Cry1Ac toxicity [13,14]. The downregulated expression of the *cadherin* gene has also been associated with resistance against the Bt Cry1Ac toxin in *Pectinophora gossypiella* [15]. Besides cadherin, the ATP-binding cassette sub-family C member 2 (ABCC2) is also recognized as an important insect molecule involved in the mode of action of Cry1A toxins [16]. Furthermore, it is known that HaCad and *Heliothis virescens* cadherin (HvCad) have a synergistic effect with ABCC2 on toxicity of Cry1A in cultured insect cells, since co-expression of cadherin receptors or the toxin-binding region of HaCad with the ABCC2 protein induced a synergistic effect on the cytotoxicity of Cry1Ac [14,16].

Even though cadherin has been shown to be an important Cry1A receptor in different Lepidopteran species, this is not always the case for some other lepidopteran insects. For instance, it has been reported that the cadherin from *Plutella xylostella* (PxCad) is not associated with resistance in *P. xylostella* to Cry1Ac [17]. However, other reports suggest that PxCad is a functional receptor of Cry1Ac, since PxCad can increase cytotoxicity of Cry1Ac when expressed in the Sf9 cell line [18,19,20]. In addition, we reported that *Spodoptera litura* cadherin (SlCad), in contrast to HaCad, cannot increase cytotoxicity of Cry1Ac when expressed in Hi5 cells, suggesting that SlCad is not a functional receptor of Cry1Ac in *S. litura* [14].

Although *S. frugiperda* is susceptible to Cry1Ab, Cry2Ab, Cry1Fa, and Vip3Aa toxins [21,22,23,24,25,26,27,28,29,30], there are no reports regarding whether *S. frugiperda* cadherin (SfCad) is involved in the mode of action of these Bt toxins. It has been shown that resistance to Cry1Fa in *S. frugiperda* is linked to different ABCC2 mutant alleles [27,28,31]. In addition, most *S. frugiperda* populations show low susceptibility to Cry1Ab or Cry1Ac toxins, in contrast to Cry1Fa that is highly active against this pest [21,32]. Here, we investigated whether SfCad is involved in the toxicity of Cry1Ab and Cry1Fa using both CRISPR/Cas 9 genome editing technology and cytotoxicity assays of Bt toxins in an insect cell line expressing SfCad. Our results suggest that *S. frugiperda* cadherin is not involved in the mode of action of Cry1Ab or Cry1Fa toxins.

## 2. Results

### 2.1. Construction of SfCad Gene Deleted Mutant by CRISPR/Cas 9 Genome Editing

**Figure 1:**
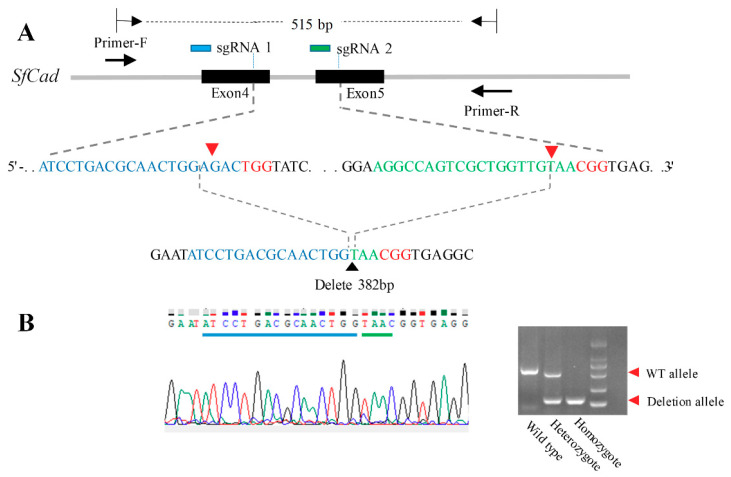
The knock-out of the *SfCad* gene. (**A**). Deleted fragment of the *SfCad* gene by the CRISPR/Cas 9 system between the two red arrow heads. (**B**). Sequencing and agarose gel electrophoresis of DNA confirming the knock-out of the *SfCad* gene.

To construct an *S. frugiperda* *cadherin* gene knockout mutant strain, we made use of the CRISPR/Cas 9 system to produce a large fragment deletion by designing two sgRNAs targeting different exons of the *SfCad* gene (Figure 1A). Freshly laid eggs were co-injected with the two *in vitro* transcribed sgRNAs, that are complementary to 20 bp DNA sequences from the fourth or fifth exons of *SfCad*, respectively, along with Cas 9 protein (Figure 1A). The results show that 22.5% (45/200) of the injected eggs hatched, and 71.1% (32) of the 45 neonates, raised in diet, survived into adults (F_0_). The F_0_ male and female moths were mass backcrossed with the DH19 strain to produce the next generation in single pair matings (F_1_).

After enough eggs were collected, genomic DNA from individual F_1_ moths was prepared. Deletion events were detected by PCR using primers across the two target site regions (Figure 1A). Fragment deletions were initially screened by agarose gel electrophoresis and then those samples that showed multiple bands were cloned using T vector, and the DNA was sequenced to identify their mutations. We found that 25% (8/32) of the examined individuals showed deletions in the *cad* gene.

From the detected mutations, we selected a 382-bp deletion to generate a homozygous knockout strain (Figure 1B). The F_1_ larvae (progeny crosses of the 382-bp deletion F_0_ moth and strain DH19) were reared to pupation, and 96 exuviates of the final instar larvae were used to prepare genomic DNA. The DNA fragments flanking the two target sites were amplified by PCR, which were 515 bp in the wild type and 133 bp in the mutant (Figure 1B). 

Among the 96 pupae screened, 30 carried the 382-bp deletion allele. Adults from these pupae were mass-crossed to obtain the F_2_ generation. The genotypes of more than 100 F_2_ individuals were visualized by agarose gel electrophoresis using gDNA samples from randomly selected exuviates of final-instar larvae. The agarose gel electrophoresis results showed that 21.4% (30/140) were homozygous for the 382-bp deletion. The 30 individuals were further sequenced and verified to be homozygotes. Finally, these homozygous individuals were pooled and mass-crossed to establish the *SfCad* knockout strain (Cad-KO).

### 2.2. Susceptibility of Cad-KO Strain to the Bt Toxins

**Figure 2:**
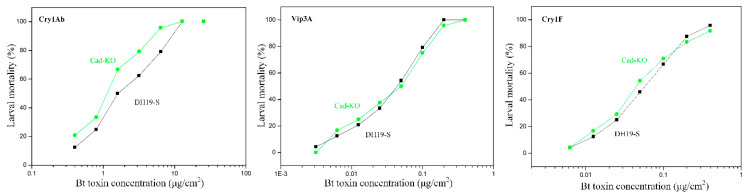
Influences of the knockout of the *SfCad* gene on susceptibility of the first instar *S. frugiperda* larvae to Cry1Ab, Vip3Aa, and Cry1Fa, respectively. Cad-KO (knockout strain), *S. frugiperda* larvae with the knockout of the *SfCad* gene. DH19-S, Bt toxin-susceptible *S. frugiperda* without knockout of the *SfCad* gene.

To determine whether *SfCad* is involved in Cry1Ab or Cry1Fa resistance in *S. frugiperda*, we performed bioassays using the Cad-KO (knockout strain) and the progenitor DH19 *S. frugiperda* strains. Bioassay results showed that the knockout strain Cad-KO did not decrease susceptibility to these two Bt toxins (Figure 2). The LC_50_ values of Cry1Ab and Cry1Fa to the Cad-KO strain were not significantly different from the control DH19 strain because their 95% fiducial limit (FL) values overlapped (Table 1), suggesting that SfCad is not a functional receptor of Cry1Ab and Cry1Fa. The bioassay data also showed that Cry1Ab was at least 20- to 40-fold less toxic to both *S. frugiperda* strains analyzed compared to Cry1Fa toxin.

As an additional control, we also tested toxicity of the Vip3Aa protein; we found that the knockout strain Cad-KO did not decrease susceptibility to the Vip3Aa toxin (Table 1 and Figure 2), indicating that SfCad does not participate in Vip3Aa toxicity.

A total of 24 larvae in each group were tested with the indicated concentrations of Bt toxins, and the values of LC_50_ were calculated after day 7 of oral feeding. Assays were done in triplicate. The 95% fiducial limits (FL) values, shown inside the parenthesis, indicate that there are no significant differences between Cad-KO and DH19-S strains in each column, since these values overlap. Cad-KO are *S. frugiperda* larvae with the knockout of the *SfCad* gene. DH19-S are Bt toxin-susceptible *S. frugiperda* larvae without knockout of the *SfCad* gene.

### 2.3. SfCAD Expression Did Not Increase Susceptibility of Hi5 Insect Cells to Cry1Ab or Cry1Fa Toxins 

**Figure 3:**
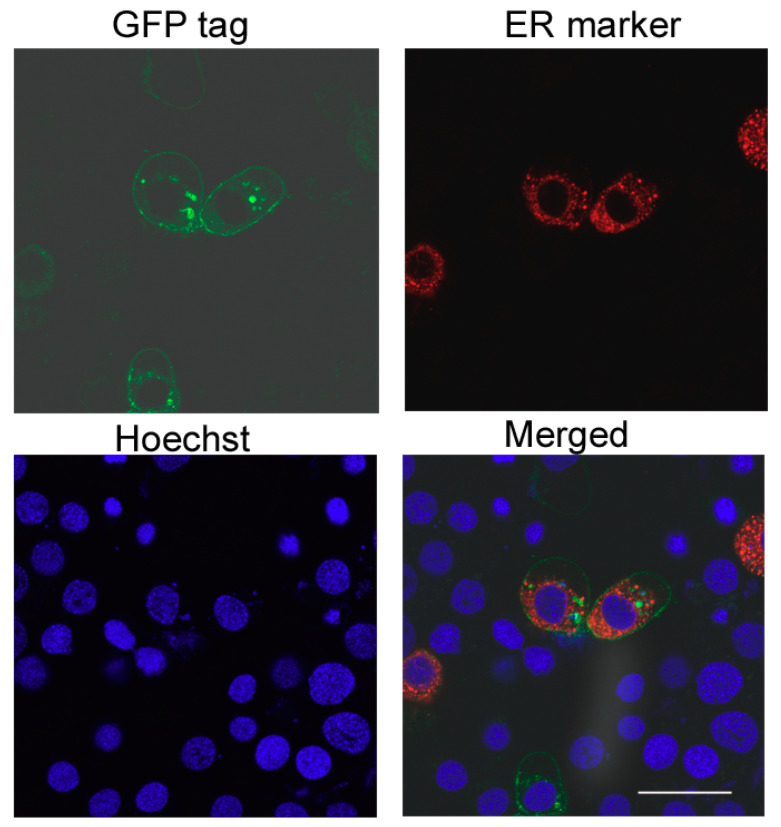
Subcellular localization of SfCad-GFP in *Trichoplusia ni* Hi5 cells. Green, SfCad-GFP (GFP tag); red, endoplasmic reticulum marker (ER marker); blue, nucleus (Hoechst). Scale bar, 50 μm.

The plasmid pIE2-SfCad-GFP was used to transiently express the fusion protein SfCad-GFP in Hi5 cells. As a control, Hi5 cells were also transfected with pIE2-SfABCC2-GFP that was previously shown to confer susceptibility to Hi5 cells to Cry1Ac toxin [33]. After transfection, cells were observed under the confocal fluorescent microscope, and the results revealed that SfCad-GFP was mainly localized on the cell membrane, suggesting proper expression and folding of the recombinant protein (Figure 3).

**Figure 4:**
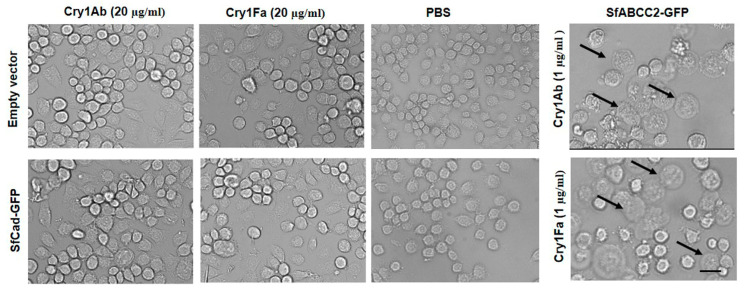
Susceptibility of Hi5 cells expressing SfCad-GFP to activated Cry1Ab and Cry1Fa toxins. The cells were transfected with plasmids pIE2-SfCad-GFP or pIE2-GFP (empty vector), respectively, and cultured for 24 h. Then, they were treated with activated toxins at 20 μg/mL for 1 h. A negative control of PBS-treated cells, treated with buffer, is included in the figure. A positive control of cells expressing SfABCC2-GFP is also shown in the figure. The susceptible cells pointed by arrow heads would become swollen, as shown in the positive control. Cells expressing SfCad-GFP or transfected with empty vector showed no swelling of the cells, similar to the negative control. Scale bar, 50 μm.

The transfection efficiency of the plasmid pIE2-SfCad-GFP was around 45–50% in Hi5 cells. Bioassay data showed that Hi5 cells expressing SfCad-GFP were still tolerant to Cry1Ab or Cry1Fa toxins, since the toxin-treated cells did not swell even at the highest concentration, 20 µg/mL, of these toxins (Figure 4 and Table 2). In contrast, Hi5 cells that were transfected with plasmid pIE2-SfABCC2 were susceptible to Cry1Ab or Cry1Fa toxins (Table 2 and Figure 4). The EC_50_ values of Bt toxins mediated by SfCad could not be calculated because there were no swollen cells after treatment with the Bt toxins for 1 h. These results also confirmed that SfCad could not mediate cytotoxicity of Cry1Ab and Cry1Fa in Hi5 cells.

### 2.4. Cry1Ac, Cry1Ab, and Cry1Fa Did Not Bind to the Toxin-Binding Region (TBR) of SfCad

**Figure 5:**
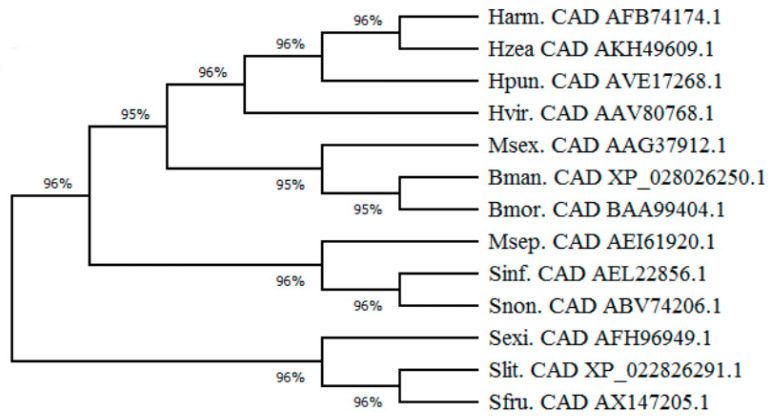
Phylogenetic analysis of cadherin protein in Lepidoptera insects. Harm, *Helicoverpa armigera*; Hzea, *Helicoverpa zea*; Hpun, *Helicoverpa punctigera*; Hvir, *Heliothis virescens*; Msex, *Manduca sexta*; Bman, *Bombyx mandarina*; Bmor, *Bombyx mori*; Msep, *Mythimna separata*; Sinf, *Sesamia inferens*; Snon, *Sesamia nonagrioides*; Sexi, *S. exigua*; Slit, *S. litura*. The Genbank accession numbers of the Cad proteins sequences used in this phylogenetic analysis are indicated in the graph.

The phylogenetic tree constructed with cadherin protein sequences from different Lepidopteran insects showed that the cadherin proteins of three *Spodoptera* species (*S. frugiperda*, *S. exigua,* and *S. litura*) cluster together and share high amino acid sequence identities (around 84%). The *Spodoptera* cadherin cluster is far away from *Helicoverpa armigera* cadherin (Figure 5). It is known that HaCad can mediate toxicity of Cry1Ac in larvae and also induces susceptibility to Cry1Ac when expressed in Hi5 cells [13,14,34].

**Figure 6:**
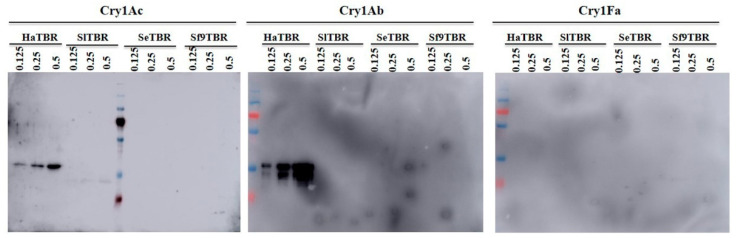
Ligand blot analysis of His-tagged toxin-binding regions (TBRs) binding to activated Cry1Ac, Cry1Ab, or Cry1Fa. The His-tagged TBRs of HaCad, SlCad, SeCad, and SlCad were used at different concentrations (0.125, 0.250, and 0.50 μg/mL) with one lane for each His-TBR. Binding of all toxins was assayed at 10 nM. Bound toxin was revealed with the corresponding polyclonal antibody (rabbit anti-Cry1Ac, rabbit anti-Cry1Ab, or rabbit anti-Cry1Fa antibody) as indicated in the Materials and Methods Section.

Finally, we performed ligand blot binding assays confirming that the toxin-binding regions (TBR) of SfCad, SeCad, and SlCad did not bind to the activated Cry1Ac, Cry1Ab, and Cry1Fa toxins, in contrast to the positive control HaCad that clearly bound to Cry1Ac- and Cry1Ab-activated toxins (Figure 6). Cry1Fa did not bind to any of the TBR regions analyzed, including the TBR from the HaCad protein (Figure 6).

## 3. Discussion

The cadherin proteins from some Lepidopteran insects are involved in susceptibility of those larvae to Cry1A toxins. Mutations or reduced expression of *cadherin* genes in *H. virescens*, *H. armigera,* or *P. gossypiella* are associated with resistance to Cry1Ac [8,11,13,35]. In addition, *Bombyx mori* cadherin was shown to be involved in toxicity of Cry1Aa and Cry1Ab toxins [36,37]. However, *S. litura* and *Trichoplusia ni* cadherins do not function as Cry1Ac receptors [14,38]. A previous study demonstrated that cadherin protein from *H. virescens* functions as a receptor for Cry1A toxins, but not for Cry1Fa, when expressed in *Drosophila* S2 cells, suggesting that Cry1A and Cry1Fa toxins may rely on different receptor molecules [39]. In the present study, both the knockout in *S. frugiperda* insect larvae and over-expression of the *SfCad* gene in cultured Hi5 insect cells indicated that SfCad is not involved in toxicity of Cry1Ab or Cry1Fa in *S. frugiperda*. As described above, it has been shown that Vip3Aa does not share receptors with Cry1A or Cry1Fa toxins [4,5,6,7]. Thus, we also performed bioassays of the Cad-KO and DH19 *S. frugiperda* strains with Vip3Aa and showed that there was also no difference in the toxicity of Vip3Aa in the two *S. frugiperda* strains (Figure 2 and Table 1). These results also show that SfCad is not a functional receptor of Vip3Aa in *S. frugiperda*.

Interestingly, we showed that expression of the *SfABCC2* transporter gene in Hi5 cells greatly increased the susceptibility to Cry1Ab or Cry1Fa toxins (Table 2), supporting that ABCC2 is a functional receptor for both Cry1Ab and Cry1Fa toxins in *S. frugiperda,* as previously reported [27]. In the case of Cry1Fa, our results agree with the fact that resistance to Cry1Fa in different populations is linked to mutant alleles of *ABCC2* [28,31]. However, the toxicity of Cry1Ab to Hi5 cells expressing SfABCC2 was 3.5-fold higher than that of Cry1Fa. These results do not correlate with the toxicities of both toxins to wild type DH19 *S. frugiperda* larvae, where Cry1Fa showed 20- to 40-fold higher toxicity than Cry1Ab (Figure 2). These results indicate that Cry1Ab toxicity is limited by some additional mechanisms, other than receptor binding, in the wild type DH19 larvae. It was reported that the lack of toxicity of Cry1Ab to an *S. frugiperda* population from México correlated with enhanced toxin degradation by midgut proteases and also with reduced receptor binding [32]. In addition, it is still possible that an additional Cry1Ab receptor is expressed in the Hi5 cells but not in *S. frugiperda* larvae. Thus, different toxin susceptibility to midgut proteases or lower binding to brush border membrane vesicles (BBMV) could explain the differences in the larval susceptibility to Cry1Fa and Cry1Ab. These hypotheses remain to be analyzed.

As mentioned above, *S. frugiperda ABCC2* mutations are linked with resistance to Cry1Fa [27,28,31]. Interestingly, some Cry1Fa-resistant *S. frugiperda* strains showed cross-resistance to Cry1Ab or Cry1Ac toxins but not to Cry2Ab or Vip3Aa toxins [20,23]. In the present study, the knockout and over-expression of the *SfCad* gene revealed that SfCad is not involved in susceptibility of *S. frugiperda* to Cry1Ab, Cry1Fa, nor Vip3Aa toxins. Nevertheless, RNAi silencing experiments of *SeCad* showed that cadherin from *S. exigua* might be involved in the toxicity of Cry1Ac and Cry2Aa to some degree [34]. Previously, we reported that SlCad did not increase the toxicity of Cry1Ac when expressed in Hi5 cells, indicating that SlCad is not a functional receptor of Cry1Ac [14]. These data agree with the lack of binding of the TBR from SlCad, SeCad, or SfCad to the Cry1Ac toxin (Figure 6) and support that cadherin proteins from the *Spodoptera* cluster species are not involved in the toxicity of Cry1Ab or Cry1Ac toxins. In the future, we will investigate whether SfCad is involved in Cry2A toxicity in *S. frugiperda*.

Even though some cadherins are not functional receptors of Cry1Ab or Cry1Ac toxins in different insect species, the cadherin protein could be a target for the evolution of Cry1Ab or Cry1Ac variants that bind to this receptor and increase their toxicity to susceptible or resistant insects where cadherin is not a functional receptor of the wild type of Cry1Ab or Cry1Ac. In the case of *T. ni,* Cry1Ac variants that could bind to the TnCad protein were selected by continuous evolution, and it was found that the Cry1Ac variants that were able to bind to TnCad were also able to counter resistance of *T. ni* insects linked to *ABCC2* mutations [40]. In addition, Cry1Ab domain III mutants were shown to increase the toxicity of Cry1Ab to different *S. frugiperda* strains, which was correlated with their increased binding to SfCad receptor [32]. Overall our results show that *S. frugiperda* cadherin is not a functional receptor of Cry1Fa and Cry1Ab toxins. Defining the structural basis for the lack of binding between Cry1Ab or Cry1Fa with SfCad could provide strategies for improving binding and toxicity of these Cry proteins to this invasive pest. 

## 4. Materials and Methods

### 4.1. S. frugiperda Strain and Insect Cell Cultures

The *S. frugiperda* strain DH19 was established from individual moths collected from Dehong, Yunnan Province of China in January 2019 and reared in laboratory conditions on artificial diet without exposure to any insecticide or Bt toxin. Insects were reared at 27 ± 2 °C and 75% ± 10% relative humidity (RH) with a photoperiod of 14L:10D. For adults, 10% sugar solution was supplied as a food source.

The *Trichoplusia ni* BTI-Tn-5B1 cell line (Hi5) was established from insect ovaries [41] and kept in our laboratory. The cell line was cultured in Grace’s insect cell culture medium (Life Technologies Co., Gand Island, NY, USA) supplemented with 10% fetal bovine serum (Life Technologies Inc.), 100 U/mL penicillin and 100 μg/mL streptomycin (Life technologies Inc.) at 28 ºC under normal atmospheric conditions.

### 4.2. Preparation of sgRNAs 

A pair of sgRNAs against the *S. frugiperda* *cadherin* gene (*SfCad*) (Genbank accession no.: AX147205.1) was designed using the sgRNAcas9 design tool [42]. The sgRNA1 target sequence (5′-ATC CTG ACG CAA CTG GAG ACT GG-3′) and sgRNA2 target sequence (5′-AGG CCA GTC GCT GGT TGT AAC GG-3′) were selected in exons 4 and 5 of the *SfCad* gene, respectively, (Figure 1A). The selected sgRNAs were analyzed in the *S. frugiperda* genome (https://bipaa.genouest.org), and no potential off-target sites were found. The DNA template for *in vitro* transcription of these sgRNAs was constructed by using PCR-based fusion of two oligonucleotides with a T7 promoter (Target F: TAA TAC GAC TCA CTA TAG + the target sequence; Target R: TTC TAG CTC TAA AAC + the reverse complementary sequence of the target). The PCR conditions were as reported by Jin et al. [43]. The sgRNAs were synthesized using an *in vitro* transcription GeneArt Precision gRNA Synthesis Kit (Thermo Fisher Scientific, Shanghai, China), according to the manufacturer’s instructions. 

### 4.3. Cas 9 Protein

Cas 9 protein (GeneArt Platinum Cas 9 Nuclease) was purchased from Thermo Fisher Scientific (Shanghai, China).

### 4.4. Egg Collection and Microinjection

Freshly laid eggs (within 2 h of oviposition) were washed with distilled water. Then, the eggs were placed on a microscope slide and fixed with double-sided adhesive tape. We injected each egg with 1–2 nL of a mixture solution containing two sgRNAs (150 ng/μL for each) and Cas 9 protein (50 ng/μL) using Nanoject III (Drummond, Broomall, PA, USA). The injected eggs were incubated at 25 °C and 65% RH for hatching.

### 4.5. Identification of SfCad Mutations Mediated by CRISPR/Cas 9 System

To identify the mutations, a specific pair of primers (Cad-F: CCT CCT CAA ATA AGA TTA CC; Cad-R: ATG ATG GGC GCA TTG TCG T) were designed that flanked the target sites, and genomic DNA samples of individual insects were used as the template. The genomic DNA of the larvae was extracted using a Multisource Genomic DNA Miniprep Kit (Axygen, New York, NY, USA) according to the manufacturer’s instructions. The PCR conditions were as reported by Jin et al. [42]. Then, 10 µL PCR products were analyzed by agarose gel electrophoresis. Multiple bands indicated that double nicking had occurred. To analyze the exact type of mutation (insertion or deletion), the bands were recovered, cloned, and sequenced by Sangon Biotech (Shanghai, China).

### 4.6. Bt Toxins and Bioassay

The activated Cry1Ab toxin and Vip3Aa protoxins used in the *in vivo* bioassay were provided by the institute of Plant Protection, Chinese Academy of Agricultural Science (CAAS), Beijing, China. The other purified activated and lyophilized Cry1Ac, Cry1Ab, and Cry1Fa toxins were kindly donated by Dr. Marianne Pusztai-Carey from Case Western Reserve University, USA. Toxicity of each Bt toxin to DH19 and SfCad knockout strain was determined with diet overlay bioassays. Gradient concentrations of Bt toxin solution were prepared by diluting the stock suspensions in PBS (pH 7.0) solution. Artificial diet (900 μL) was dispensed into a 24-well plate (surface area per well = 2 cm^2^) and after the diet cooled down, 50 μL Bt toxin solution was applied on the surface in each well. A single 1st-instar larva was put in each well after the toxin solution was dried at room temperature, and mortality was recorded after 7 days. The LC_50_ (median lethal concentration that killed 50% of the tested larvae) and the corresponding 95% fiducial limits were calculated through Probit analysis of the mortality data using SPSS. Control wells were treated with buffer solution.

### 4.7. Plasmids, Transfection, and Fluorescence Observation

The *SfCad* gene was synthesized by Genescript Company (Nanjing, China) and inserted into a pGEM-T easy vector (Promega Inc., Madison, WI, USA). Then, the gene was amplified by PCR and inserted into pIE2-GFP-N1, and the new construct was named as pIE2-SfCad-GFP [14]. The plasmid purified from the transformed *E. coli* DH5α was transfected into Hi5 cells as previously reported [14]. Briefly, Hi5 cells were grown overnight in 48-well cell culture plates (Corning Inc., New York, NY, USA) at 1.2 × 10^5^ cells/well. Then, the transfection was performed using the mixture of the plasmid (250 ng/well) with a transfection kit Genefusion HD (1 μL/well) (Promega Inc., Madison, WI, USA). Plasmids pIE2-GFP-N1, pIE2-SfCad-GFP, pIE2-SfABCC2-GFP, and pIE2-dsRED-ER were previously reported [33,44]. At 24 h post transfection, the cells were fixed using 4% paraformaldehyde for 10 min, and stained by Hoechst 33,342 (1 μg/mL) for 10 min. Then, the cells were observed and photographed under a laser confocal scanning microscope (Carl Zeiss, Jena Deutschland, Germany). The transfection efficiency was calculated: A = the number of cells emitting green fluorescence (SfCad-GFP) divided by the number of cells emitting blue fluorescence (nucleus stained by Hoechst 33,342) × 100%. Three biological replicates were performed.

### 4.8. Cytotoxicity Assay 

Hi5 cells were transfected using single plasmids (pIE2-SfCad-GFP or pIE2-SfABCC2-GFP) as described above. At 24 h post transfection, the cells were treated with the indicated toxin concentrations (at least five different concentrations, two-fold serial dilution) of activated Bt toxins (Cry1Ab or Cry1Fa) for 1 h, and they were photographed under an inverted confocal microscope (Nikon, Tokyo, Japan). The cells transfected with the empty vector (pIE2-GFP-N1) were used as a control group and were also treated with the Cry toxins. An additional negative control of cells treated with phosphate buffer solution (PBS) was included in these assays. The percentage of swollen cells resulting from these toxin treatments was calculated as follows: B = the number of the swollen cells divided by the number of the total cells × 100%. The percentage of the swollen cells expressing SfCad-GFP or SfABCC2-GFP was calculated as follows: C = B/A × 100%. The transfection efficiency (A) was described above in Section 4.7. The effective concentration inducing 50% mortality value (EC_50_) was obtained by regression analysis using SPPS 16.0 software. For two particular populations, the EC_50_ values were considered as significantly different if their 95% fiducial limits (FL) did not overlap [45].

### 4.9. Construction of a Lepidoptera Cadherin Phylogenetic Tree

The sequences of Lepidopteran insect cadherin proteins were selected for constructing a cadherin evolutionary tree by analyzing their phylogeny. GenBank accession numbers of the sequences of these cadherin proteins are as follow. Harm: *Helicoverpa armigera* cadherin, AFB74174.1; Hzea: *Helicoverpa zea* cadherin, AKH49609.1; Hpun: *Helicoverpa punctigera* cadherin, AVE17268.1; Hvir: *Heliothis virescens* cadherin, AAV80768.1; Msex: *Manduca sexta* cadherin, AAG37912.1; Bman: *Bombyx mandarina* cadherin, XP_028026250.1; Bmor: *Bombyx mori* cadherin, BAA99404.1; Msep: *Mythimna separata* cadherin, AEI61920.1; Sinf: *Sesamia inferens* cadherin, AEL22856.1; Snon: *Sesamia nonagrioides* cadherin, ABV74206.1; Sexi: *S. exigua* cadherin, AFH96949.1; Slit: *S. litura* cadherin, XP_022826291.1. The phylogeny of these sequences was analyzed using the neighbor-joining tree method with MEGA 5.0 software (https://mega.software.informer.com/5.0/).

### 4.10. Purification of Proteins Expressed in Bacteria

The coding DNA of toxin-binding regions of *SfCad*, *SlCad*, *SeCad,* and *HaCad* were amplified by PCR from the corresponding plasmids containing these genes or the cDNA obtained from midgut tissue of these insects [14]. The primers are listed in Table 3. The amplified fragments were purified and digested with restriction nucleases and cloned into the cleaved plasmids listed in Table 3. The constructs were transformed into *Escherichia coli* BL21 cells and the His-tagged proteins were purified with Ni-NTA affinity column (GE Healthcare Bioscience, Piscataway, NJ, USA) according to the manufacturer’s manual. Detailed protocols were described previously [14]. All the purified proteins were stored at −80 °C until use.

### 4.11. Ligand Blot Assays

The 6 × His-tagged HaCad, SfCad, SlCad, or SeCad TBRs were separated on 12% SDS-PAGE gel and transferred onto Polyvinylidene fluoride (PVDF) membrane (three different membranes were prepared). The loading of proteins on these membranes was checked by Ponceau S staining 0.2% (*w*/*v*) in 3% (*v*/*v*) acetic acid and followed by complete destaining by washing with water. These membranes were blocked with 2% BSA in PBS-Tween (0.2%) for 3 h, then each membrane was incubated with a different activated Cry toxin (Cry1Ab, Cry1Ac or Cry1Fa toxin) at 10 nM for 2 h. After washing three times with PBS-Tween (0.2%), the membranes were further incubated with the corresponding polyclonal antibody (rabbit anti-Cry1Ac, rabbit anti-Cry1Ab, or rabbit anti-Cry1Fa antibody) diluted in PBS-Tween (0.2%) at 1:1000 dilution for 3 h. Then, the membranes were incubated with horseradish peroxidase (HRP)-conjugated goat anti-rabbit secondary antibody (Abbkine) diluted in PBS at 1:10,000. Finally, the membranes were incubated with the enhanced chemiluminescence (ECL) reagent (GE Healthcare Biosciences, Piscataway, NJ, USA) and then covered with X-ray film for exposure for a few minutes, and the film was developed and fixed as previously described [46].

## Figures and Tables

**Table 1:** Comparison of the susceptibility of first instar *S. frugiperda* larvae from the Cad-KO and DH19-S strains to different Bt toxins.

Strain.	Cry1Ab LC_50_ in μg/cm^2^ (95% of FL)	Cry1Fa LC_50_ in μg/cm^2^ (95% of FL)	Vip3Aa LC_50_ in μg/cm^2^ (95% of FL)
Cad-KO	1.103 (0.798–1.453)	0.05 (0.037–0.069)	0.035 (0.026–0.047)
DH19-S	1.797 (1.311–2.458)	0.054 (0.041–0.073)	0.033 (0.025–0.044)

**Table 2:** Effect of SfCad or SfABCC2 on the cytotoxicity of activated Cry1Ab and Cry1Fa toxins in Hi5 cells.

Toxin	Putative Receptor	EC_50_ (µg/mL)	95% FL	Slope ± SE	χ^2^	df
Cry1Ab	SfCAD-GFP	— *	—	—	—	—
Cry1Ab	SfABCC2-GFP	0.06_a_ **	0.04–0.08	1.55 ± 0.08	7.20	3
Cry1Fa	SfCAD-GFP	—	—	—	—	—
Cry1Fa	SfABCC2-GFP	0.23_b_	0.19–0.27	2.18 ± 0.09	6.77	3

* indicates that the cells expressing the putative receptors are not susceptible to the indicated toxins; ** the different lowercase letters indicate that there are significant differences between EC_50_ values of Cry1Ab and Cry1Fa in the same column.

**Table 3:** Primers used for expression of the different fragments of proteins.

Fragment	Forward Primer (5′–3′)	Reverse Primer (5′–3′)	Vector
His-HaTBR	CCGGAATTCTACGATTCGTGCTACGGACGGT	CCCAAGCTTCAGGTACACCTTCACTTCCGT-3	pET22b (Novagen, Madison, WI, USA)
His-SeTBR	CCGGAATTCTGTTATCCGAGCTACTGATGG	CCCAAGCTTCATGAAGATTGTCACTTCAGCTCGATC	pET22b
His-SlTBR	CCGGAATTCTGTTATTCGTGCCACGGATGGT	CCCAAGCTTCATGTAGATTATAACTTTTGCTCG	pET22b
His-SfTBR	CCGGAATTCTGGAGGCGGTGGAGGCGGTGTTATTCGGGCCACGGACGGCG	CCCAAGCTTCATGTAAATTGACACTTTTGCTCGATCACTCGC	pET22b

The underlined letters indicate restriction sites of endonucleases.
